# Ageing as older Chinese immigrants in Europe—a qualitative systematic literature review considering perspectives from older immigrants, relatives, and professionals

**DOI:** 10.3389/fpubh.2025.1492356

**Published:** 2025-04-07

**Authors:** Hongxuan Xu, Sigrid Stjernswärd, Stinne Glasdam, Ragnhild Julante Andersen Gulestø, Cong Fu

**Affiliations:** ^1^Department of Care Science, Faculty of Health and Society, Malmö University, Malmö, Sweden; ^2^Department of Health Sciences, Faculty of Medicine, Lund University, Lund, Sweden; ^3^Faculty of Health Science, VID Specialized University, Oslo, Norway

**Keywords:** ageing, Chinese immigrants, Europe, everyday life, older adults, qualitative literature review

## Abstract

**Introduction:**

As the Chinese immigrant population in European countries ages, it is important to gain a deeper understanding of Chinese immigrants’ ageing processes from a life course perspective by recognising the complex interactions between social, cultural, and institutional constructs and dynamics. This article aimed at exploring how older Chinese immigrants in Europe handle everyday lives in respect to ageing from the perspectives of older Chinese immigrants, their relatives, and health/social care professionals.

**Methods:**

The study is registered in PROSPERO (CRD42023455411), and the PRISMA 2020 checklist guided the study. A qualitative systematic review was conducted through searches in the databases CINAHL, Embase, PsycINFO, Medline/PubMed, SocINDEX, Web of Science, and pearl search in Scopus (last search 3 September, 2023). Inclusion criteria were: (1) Studies about Chinese immigrants’ everyday lives, living, and ageing, or studies focusing on their use of health/social care services, (2) Perspectives of Chinese immigrants in Europe, their relatives, and related health/social care professionals, (3) Qualitative peer- reviewed studies published in English, and (4) publications from 2000–2023. The initial search retrieved 842 publications. Seventeen publications were included and analysed through a thematic analysis.

**Results:**

The results presented the included studies’ characteristics and four themes: *Everyday life as an older adult mirrored the life lived*, *Work and working conditions as significant for ageing*, *Cultural complexes that shape social identities, Immigrants’ social position as significant for encounters with health and social care professionals*.

**Discussion:**

Older Chinese immigrants’ everyday lives related to ageing were not only dynamically influenced by social, interpersonal, and institutional factors accumulated in their life trajectories, but reflected the process of constructing social and cultural identity in their new homeland. Future policies should promote culturally responsive healthcare, social services, and employment support to address the unique ageing experiences of older Chinese immigrants.

**Systematic review registrations:**

The systematic review has been registered on PROSPERO and the registration number is CRD42023455411.

## Introduction

International migration has increased over the past few decades (1). Worldwide, Chinese immigrants constituted the third largest population of foreign-born immigrants in 2019 (2). Europe is the largest international migration destination, with 87 million immigrants in 2020, of which 4.1% were people 65+ years (2). The immigration from China to Europe dates back to the 19th century. By the end of 2021, around 2.45 million people of Chinese birth or descent were living in Europe (3). Immigration patterns from China to Europe are characterised by different waves, for instance, labour immigration, education, and family reunification (4, 5). As the Chinese immigrant population ages, it seems important to focus on their experiences and daily routines as they navigate ageing in a new home country to understand their life situations and be able to offer appropriate health/social care support. Everyday life in the current article refers to the multidimensional processes in which people act, think, and feel, often repeatedly on a daily basis, taking shape within the family, work, or living environment and constituting the structural conditions of life (6). The life course perspective offers a way to understand how chronological age, life transitions, social relationships and the environment shape people’s lives, health status and health trajectories (7–10). In the current review, it serves as an additional lens to understand the complexity and diversity of the lives of older Chinese immigrants, not least in light of the potential implications for health and social care workers and systems and the tailoring of supportive interventions for the population at stake (9).

At present, European countries with significant immigration are experiencing accelerated population ageing compared to other regions of the world (2). The ageing pathways of immigrants vary across European countries due to the diversity of countries’ immigration histories, heterogeneity of immigrants’ life courses, and immigration trajectories. Immigration can be regarded as a distinctive life event with implications for the remainder of the life course. This in turn reflects that variations in life experiences may lead to different cognitions and experiences of ageing (11). Individualistic cultures in western countries stress personal attributes and individualistic values of a person with loose ties with other members (12). Contrarily, in some Asian countries and particularly in China, collectivist cultures emphasise interdependency and group interests (13). Hence, culture can be a crucial factor in shaping people’s attitudes towards ageing, older adult care, and related health-seeking behaviors (14, 15). However, while cultural determinants undeniably influence people’s attitudes and perceptions, limiting perspectives to these factors alone is overly simplistic (16). To gain a more profound understanding of the ageing processes among older adults from immigrant backgrounds outside of Europe, it is crucial to recognise the complex interplay of diverse social constructs and dynamics, such as social class, gender, and disability (16).

Most immigrants face the process of culturally integrating into the new homeland’s environment, which may result in different consequences, that is, cultural harmony and conflicts (11). Yet the process of acculturation to a western lifestyle has a significant impact on lives and daily living for many (especially older) immigrants (17). Both older adults who immigrate at older ages and those who have lived and grown old in new homelands, including family members, may undergo a range of challenges of ageing in a foreign country (11). Immigration as a major life event entails a variety of considerable changes in cultural opportunities, living environment, employment, and social relationships (18). Social relationships encompass social networks and supportive social connections, while immigrants’ ability to form and maintain social relationships varies with contextual factors such as immigration age and acculturation level (19). van Dyk et al. (20) show that older adults’ lifestyle choices keep their ailments ‘at arm’s length’, where the freedom of retirement was regarded as a valued part of their ageless identity, framing ageing as a continuum of life into older years. Age and ageing can also be associated with different metaphors, lifestyles, and prerequisites influenced by individuals’ social position, interests, and health status (21). Views on age and ageing are influenced by the ways individuals, societies, and different parties in society express themselves about these subjects (21). Multiple transitions take place in one’s late life, each of which signifies a process of ending or disengagement into a new beginning in search of meaning (22). Transitions disrupt known connections and lose familiar points of reference, which often results in older people feeling marginalised in the world they live in (23). Having to grow old in the host country further amplifies the transition. In healthy transitions, the older immigrants’ lives are often restructured in a predictable, manageable, and pleasurable way. However, the sustained daily routines may no longer work in the new situation, possibly leading to instability, disorder, and emptiness in their everyday life (22).

The Chinese cultural norm of filial piety often places younger generations under an obligation to care for older family members, which may hinder older Chinese immigrants’ use of formal health services such as nursing homes and homecare (24–26). Deeply rooted collectivist values may result in changing information and concealing the truth to protect the family’s collective interests (27). In some European countries, e.g., the Netherlands, Sweden, Norway, and Denmark, older immigrants appear to use less health/social care services than most of the population and often experience worse health outcomes such as poor self-rated health, diminished work capacity, and physical disabilities than the host populations (28–31). Studies also highlight structural barriers to health/social care service utilisation, suggesting that lower degree of service use among older immigrants and their families may also be attributed to insufficient care provision and unmet individual facilitation (32–34). Furthermore, Chinese immigrants’ health beliefs influenced by traditional Chinese philosophy may also shape their perceptions and responses to ageing, dying and death, health, and illness (35, 36). Studies find that Chinese immigrants are often reluctant to use advanced care planning and were thus less likely, like other minority groups, to receive formal palliative care services than host populations (37, 38). In addition, older Chinese immigrants residing in nursing homes face adaptation challenges in relation to western cultures and healthcare systems (39). Understanding people’s culturally relevant health perspectives and needs is crucial to provide respectful and effective culturally congruent care (36).

Despite the growing population of older Chinese immigrants in Europe, research attention on their experiences remains limited compared to studies conducted in North America and Australia. The distinct migration policies, welfare systems, and healthcare structures in Europe may influence ageing experiences of older immigrants in ways that differ from those in other regions. Additionally, there are limited qualitative reviews focusing on older Chinese immigrants in Europe. This review aimed at exploring how older Chinese immigrants in Europe handle everyday lives in respect to ageing and health from the perspectives of older Chinese immigrants, their relatives, and health/social care professionals.

## Materials and methods

This qualitative systematic review synthesised findings from qualitative studies to obtain a comprehensive understanding of older Chinese immigrants’, their relatives’, and health/social care professionals’ perspectives on older Chinese immigrants’ everyday lives. The Preferred Reporting Items for Systematic Reviews and Meta-Analyses (PRISMA) recommendations (109) was used to support the current article. The review protocol was registered in PROSPERO (2023/no Blinded for reviewers).

### Search strategy

A primary search was conducted in the databases CINAHL, Embase, PsycINFO, Medline by the use of the search engine PubMed, SocINDEX, and Web of Science in September 2023 (last search 3 September) (see Table 1 for keywords and search strategies). The search terms were developed according to the population (P), exposure (E), and outcome (O) (PEO) model. The search was limited to peer-reviewed qualitative articles, written in English, and published from 2000 to 2023. Studies written in languages other than English were excluded due to feasibility constraints, such as resource limitations for translation and the risk of misinterpretation. A citation pearl search was conducted in Scopus, 3 September 2023.

**Table 1:** Keywords and search strategies.

Search terms		Outcomes
PubMed		
1#	“Aged”[Mesh] OR Elder OR older OR senior OR old people OR aged OR older adult OR pensioner*	666,0786
2#	(Chinese[Title/Abstract] OR china[Title/Abstract]) AND (migrant*[Title/Abstract] OR immigrant*[Title/Abstract] OR diaspora[Title/Abstract])	4,339
3#	Sweden[Title/Abstract] OR Swedish[Title/Abstract] OR Norway[Title/Abstract] OR Norwegian*[Title/Abstract] OR Denmark[Title/Abstract] OR Danish[Title/Abstract] OR Dane*[Title/Abstract] OR Scandinavia[Title/Abstract] OR Scandinavian*[Title/Abstract] OR Finland[Title/Abstract] OR Finnish[Title/Abstract] OR Iceland[Title/Abstract] OR Icelandic[Title/Abstract] OR United Kingdom[Title/Abstract] OR British[Title/Abstract] OR Brit[Title/Abstract] OR Brits[Title/Abstract] OR England[Title/Abstract] OR English[Title/Abstract] OR Wales[Title/Abstract] OR Welch[Title/Abstract] OR Scotland[Title/Abstract] OR Scot[Title/Abstract] OR Scots[Title/Abstract] OR Ireland[Title/Abstract] OR Irish[Title/Abstract] OR Germany[Title/Abstract] OR German*[Title/Abstract] OR Holland[Title/Abstract] OR Netherlands[Title/Abstract] OR Dutch[Title/Abstract] OR Belgium[Title/Abstract] OR Belgian*[Title/Abstract] OR Luxemburg*[Title/Abstract] OR Benelux[Title/Abstract] OR France[Title/Abstract] OR French[Title/Abstract] OR Spain[Title/Abstract] OR Spanish[Title/Abstract] OR Andorra[Title/Abstract] OR Andorran*[Title/Abstract] OR Portugal[Title/Abstract] OR Portuguese[Title/Abstract] OR Italy[Title/Abstract] OR Italian*[Title/Abstract] OR San Marino[Title/Abstract] OR Malta[Title/Abstract] OR Maltese[Title/Abstract] OR Switzerland[Title/Abstract] OR Swiss[Title/Abstract] OR Austria[Title/Abstract] OR Austrian[Title/Abstract] OR Liechtenstein[Title/Abstract] OR Czech[Title/Abstract] OR Slovakia[Title/Abstract] OR Slovakian*[Title/Abstract] OR Monaco[Title/Abstract] OR Monegasque*[Title/Abstract] OR Estonia[Title/Abstract] OR Estonian*[Title/Abstract] OR Latvia[Title/Abstract] OR Latvian*[Title/Abstract] OR Lithuania[Title/Abstract] OR Lithuanian*[Title/Abstract] OR Baltic[Title/Abstract] OR Hungary[Title/Abstract] OR Hungarian*[Title/Abstract] OR Poland[Title/Abstract] OR Polish[Title/Abstract] OR Russia[Title/Abstract] OR Russian*[Title/Abstract] OR Ukrain*[Title/Abstract] OR Belarus[Title/Abstract] OR Moldova[Title/Abstract] OR Moldovan*[Title/Abstract] OR Armenia[Title/Abstract] OR Armenian*[Title/Abstract] OR Romania[Title/Abstract] OR Romanian*[Title/Abstract] OR Bulgaria[Title/Abstract] OR Bulgarian*[Title/Abstract] OR Greece[Title/Abstract] OR Greek*[Title/Abstract] OR Albania[Title/Abstract] OR Albanian*[Title/Abstract] OR Balkan*[Title/Abstract] OR Slovenia[Title/Abstract] OR Slovenian*[Title/Abstract] OR Croatia[Title/Abstract] OR Croatian*[Title/Abstract] OR Macedonia[Title/Abstract] OR Macedonian*[Title/Abstract] OR Serbia[Title/Abstract] OR Serbian*[Title/Abstract] OR Montenegro[Title/Abstract] OR Montenegrin*[Title/Abstract] OR Kosovo*[Title/Abstract] OR Mediterranean*[Title/Abstract] OR Europe[Title/Abstract] OR European*[Title/Abstract] OR “the EU”[Title/Abstract]	1,703,960
4#	end-of-life OR palliative OR dying OR pension OR care OR ageing OR illness OR frailty OR disease*	11,540,981
	1# AND 2# AND 3# AND 4#	228
Filter(s)	Published Date: 2000–2023	206
Embase		
1#	‘aged’/exp. OR elder OR older OR senior OR ‘old people’ OR aged OR older adult OR pensioner*	6,128,588
2#	‘chinese’/exp. OR ‘china’/exp. OR chinese:ti,ab,kw OR china:ti,ab,kw	691,741
3#	migrant*:ti,ab,kw OR immigrant*:ti,ab,kw OR diaspora:ti,ab,kw	58,332
4#	sweden:ti,ab,kw OR swedish:ti,ab,kw OR swede*:ti,ab,kw OR norway:ti,ab,kw OR norwegian*:ti,ab,kw OR denmark:ti,ab,kw OR danish:ti,ab,kw OR dane*:ti,ab,kw OR scandinavia:ti,ab,kw OR scandinavian*:ti,ab,kw OR finland:ti,ab,kw OR finnish:ti,ab,kw OR iceland:ti,ab,kw OR icelandic:ti,ab,kw OR ‘united kingdom’:ti,ab,kw OR british:ti,ab,kw OR brit:ti,ab,kw OR brits:ti,ab,kw OR england:ti,ab,kw OR english:ti,ab,kw OR wales:ti,ab,kw OR welch:ti,ab,kw OR scotland:ti,ab,kw OR scot:ti,ab,kw OR scots:ti,ab,kw OR ireland:ti,ab,kw OR irish:ti,ab,kw OR germany:ti,ab,kw OR german*:ti,ab,kw OR holland:ti,ab,kw OR netherlands:ti,ab,kw OR dutch:ti,ab,kw OR belgium:ti,ab,kw OR belgian*:ti,ab,kw OR luxemburg*:ti,ab,kw OR benelux:ti,ab,kw OR france:ti,ab,kw OR french:ti,ab,kw OR spain:ti,ab,kw OR spanish:ti,ab,kw OR andorra:ti,ab,kw OR andorran*:ti,ab,kw OR portugal:ti,ab,kw OR portuguese:ti,ab,kw OR italy:ti,ab,kw OR italian*:ti,ab,kw OR ‘san marino’:ti,ab,kw OR malta:ti,ab,kw OR maltese:ti,ab,kw OR switzerland:ti,ab,kw OR swiss:ti,ab,kw OR austria:ti,ab,kw OR austrian:ti,ab,kw OR liechtenstein:ti,ab,kw OR czech:ti,ab,kw OR slovakia:ti,ab,kw OR slovakian*:ti,ab,kw OR monaco:ti,ab,kw OR monegasque*:ti,ab,kw OR estonia:ti,ab,kw OR estonian*:ti,ab,kw OR latvia:ti,ab,kw OR latvian*:ti,ab,kw OR lithuania:ti,ab,kw OR lithuanian*:ti,ab,kw OR baltic:ti,ab,kw OR hungary:ti,ab,kw OR hungarian*:ti,ab,kw OR poland:ti,ab,kw OR polish:ti,ab,kw OR russia:ti,ab,kw OR russian*:ti,ab,kw OR ukrain*:ti,ab,kw OR belarus:ti,ab,kw OR moldova:ti,ab,kw OR moldovan*:ti,ab,kw OR armenia:ti,ab,kw OR armenian*:ti,ab,kw OR romania:ti,ab,kw OR romanian*:ti,ab,kw OR bulgaria:ti,ab,kw OR bulgarian*:ti,ab,kw OR greece:ti,ab,kw OR greek*:ti,ab,kw OR albania:ti,ab,kw OR albanian*:ti,ab,kw OR balkan*:ti,ab,kw OR slovenia:ti,ab,kw OR slovenian*:ti,ab,kw OR croatia:ti,ab,kw OR croatian*:ti,ab,kw OR macedonia:ti,ab,kw OR macedonian*:ti,ab,kw OR serbia:ti,ab,kw OR serbian*:ti,ab,kw OR montenegro:ti,ab,kw OR montenegrin*:ti,ab,kw OR kosovo*:ti,ab,kw OR mediterranean*:ti,ab,kw OR europe:ti,ab,kw OR european*:ti,ab,kw OR ‘the eu’:ti,ab,kw	2,659,709
5#	‘end of life’/exp. OR ‘end of life’ OR palliative OR ‘dying’/exp. OR dying OR ‘pension’/exp. OR pension OR ‘care’/exp. OR care OR ‘ageing’/exp. OR ageing OR ‘illness’/exp. OR illness OR ‘frailty’/exp. OR frailty OR disease*	29,817,780
6#	1# AND 2# AND 3# AND 4#AND 5#	371
#7	#6 AND (2000:py OR 2001:py OR 2002:py OR 2003:py OR 2004:py OR 2005:py OR 2006:py OR 2007:py OR 2008:py OR 2009:py OR 2010:py OR 2011:py OR 2012:py OR 2013:py OR 2014:py OR 2015:py OR 2016:py OR 2017:py OR 2018:py OR 2019:py OR 2020:py OR 2021:py OR 2022:py OR 2023:py)	346
CINAHL		
1#	(MH “Aged+”) OR (Elder OR older OR senior OR old people OR aged OR older adult OR pensioner*)	1,241,452
2#	(MH “Immigrants+”) OR TI (migrant* OR immigrant* OR diaspora) OR AB (migrant* OR immigrant* OR diaspora)	30,213
3#	TI (chinese OR china) OR AB (chinese OR china)	107,141
4#	TI (Sweden OR Swedish OR Swede* OR Norway OR Norwegian* OR Denmark OR Danish OR Dane* OR Scandinavia OR Scandinavian* OR Finland OR Finnish OR Iceland OR Icelandic OR United kingdom OR British OR Brit OR Brits OR England OR English OR Wales OR Welch OR Scotland OR Scot OR Scots OR Ireland OR Irish OR Germany OR German* OR Holland OR Netherlands OR Dutch OR Belgium OR Belgian* OR Luxemburg* OR Benelux OR France OR French OR Spain OR Spanish OR Andorra OR Andorran* OR Portugal OR Portuguese OR Italy OR Italian* OR San Marino OR Malta OR Maltese OR Switzerland OR Swiss OR Austria OR Austrian* OR Liechtenstein OR Czech OR Slovakia OR Slovakian* OR Monaco OR Monegasque* OR Estonia OR Estonian* OR Latvia OR Latvian* OR Lithuania OR Lithuanian* OR Baltic OR Hungary OR Hungarian* OR Poland OR Polish OR Russia OR Russian* OR Ukrain* OR Belarus OR Moldova OR Moldovan* OR Armenia OR Armenian* OR Romania OR Romanian* OR Bulgaria OR Bulgarian* OR Greece OR Greek* OR Albania OR Albanian* OR Balkan* OR Slovenia OR Slovenian* OR Croatia OR Croatian* OR Macedonia OR Macedonian* OR Serbia OR Serbian* OR Montenegro OR Montenegrin* OR Kosovo* OR Mediterranean* OR Europe OR European* OR “the EU”) OR AB (Sweden OR Swedish OR Swede* OR Norway OR Norwegian* OR Denmark OR Danish OR Dane* OR Scandinavia OR Scandinavian* OR Finland OR Finnish OR Iceland OR Icelandic OR United kingdom OR British OR Brit OR Brits OR England OR English OR Wales OR Welch OR Scotland OR Scot OR Scots OR Ireland OR Irish OR Germany OR German* OR Holland OR Netherlands OR Dutch OR Belgium OR Belgian* OR Luxemburg* OR Benelux OR France OR French OR Spain OR Spanish OR Andorra OR Andorran* OR Portugal OR Portuguese OR Italy OR Italian* OR San Marino OR Malta OR Maltese OR Switzerland OR Swiss OR Austria OR Austrian* OR Liechtenstein OR Czech OR Slovakia OR Slovakian* OR Monaco OR Monegasque* OR Estonia OR Estonian* OR Latvia OR Latvian* OR Lithuania OR Lithuanian* OR Baltic OR Hungary OR Hungarian* OR Poland OR Polish OR Russia OR Russian* OR Ukrain* OR Belarus OR Moldova OR Moldovan* OR Armenia OR Armenian* OR Romania OR Romanian* OR Bulgaria OR Bulgarian* OR Greece OR Greek* OR Albania OR Albanian* OR Balkan* OR Slovenia OR Slovenian* OR Croatia OR Croatian* OR Macedonia OR Macedonian* OR Serbia OR Serbian* OR Montenegro OR Montenegrin* OR Kosovo* OR Mediterranean* OR Europe OR European* OR “the EU”	537,762
5#	(end-of-life OR palliative OR dying OR pension OR care OR ageing OR illness OR frailty OR disease*) OR (end-of-life OR palliative OR dying OR pension OR care OR ageing OR illness OR frailty OR disease*)	2,983,626
6#	1# AND 2# AND 3# AND 4#AND 5#	104
Limiters	Published Date: 20000101–20,231,231	97
PsycINFO		
1#	MA aged OR (Elder OR older OR senior OR old people OR aged OR older adult OR pensioner*)	821,570
2#	TI (chinese OR china) OR AB (chinese OR china)	81,300
3#	TI (migrant* OR immigrant* OR diaspora) OR AB (migrant* OR immigrant* OR diaspora)	42,061
4#	TI (Sweden OR Swedish OR Swede* OR Norway OR Norwegian* OR Denmark OR Danish OR Dane* OR Scandinavia OR Scandinavian* OR Finland OR Finnish OR Iceland OR Icelandic OR United kingdom OR British OR Brit OR Brits OR England OR English OR Wales OR Welch OR Scotland OR Scot OR Scots OR Ireland OR Irish OR Germany OR German* OR Holland OR Netherlands OR Dutch OR Belgium OR Belgian* OR Luxemburg* OR Benelux OR France OR French OR Spain OR Spanish OR Andorra OR Andorran* OR Portugal OR Portuguese OR Italy OR Italian* OR San Marino OR Malta OR Maltese OR Switzerland OR Swiss OR Austria OR Austrian* OR Liechtenstein OR Czech OR Slovakia OR Slovakian* OR Monaco OR Monegasque* OR Estonia OR Estonian* OR Latvia OR Latvian* OR Lithuania OR Lithuanian* OR Baltic OR Hungary OR Hungarian* OR Poland OR Polish OR Russia OR Russian* OR Ukrain* OR Belarus OR Moldova OR Moldovan* OR Armenia OR Armenian* OR Romania OR Romanian* OR Bulgaria OR Bulgarian* OR Greece OR Greek* OR Albania OR Albanian* OR Balkan* OR Slovenia OR Slovenian* OR Croatia OR Croatian* OR Macedonia OR Macedonian* OR Serbia OR Serbian* OR Montenegro OR Montenegrin* OR Kosovo* OR Mediterranean* OR Europe OR European* OR “the EU”) OR AB (Sweden OR Swedish OR Swede* OR Norway OR Norwegian* OR Denmark OR Danish OR Dane* OR Scandinavia OR Scandinavian* OR Finland OR Finnish OR Iceland OR Icelandic OR United kingdom OR British OR Brit OR Brits OR England OR English OR Wales OR Welch OR Scotland OR Scot OR Scots OR Ireland OR Irish OR Germany OR German* OR Holland OR Netherlands OR Dutch OR Belgium OR Belgian* OR Luxemburg* OR Benelux OR France OR French OR Spain OR Spanish OR Andorra OR Andorran* OR Portugal OR Portuguese OR Italy OR Italian* OR San Marino OR Malta OR Maltese OR Switzerland OR Swiss OR Austria OR Austrian* OR Liechtenstein OR Czech OR Slovakia OR Slovakian* OR Monaco OR Monegasque* OR Estonia OR Estonian* OR Latvia OR Latvian* OR Lithuania OR Lithuanian* OR Baltic OR Hungary OR Hungarian* OR Poland OR Polish OR Russia OR Russian* OR Ukrain* OR Belarus OR Moldova OR Moldovan* OR Armenia OR Armenian* OR Romania OR Romanian* OR Bulgaria OR Bulgarian* OR Greece OR Greek* OR Albania OR Albanian* OR Balkan* OR Slovenia OR Slovenian* OR Croatia OR Croatian* OR Macedonia OR Macedonian* OR Serbia OR Serbian* OR Montenegro OR Montenegrin* OR Kosovo* OR Mediterranean* OR Europe OR European* OR “the EU”	5,143,411
5#	end-of-life OR palliative OR dying OR pension OR care OR ageing OR illness OR frailty OR disease*	292,967
6#	1# AND 2# AND 3# AND 4#AND 5#	583
Limiters	Year of Publication: 2000–2023	556
SocINDEX		
1#	DE “OLDER people” OR (Elder OR older OR senior OR old people OR aged OR older adult OR pensioner*)	152,448
2#	((DE “HEALTH of immigrants”) OR (DE “MEDICAL care of immigrants”)) OR TI (immigrant* OR migrant* OR diaspora) OR AB (immigrant* OR migrant* OR diaspora)	59,116
3#	TI (chinese OR china) OR AB (chinese OR china)	56,296
4#	TI (Sweden OR Swedish OR Swede* OR Norway OR Norwegian* OR Denmark OR Danish OR Dane* OR Scandinavia OR Scandinavian* OR Finland OR Finnish OR Iceland OR Icelandic OR United kingdom OR British OR Brit OR Brits OR England OR English OR Wales OR Welch OR Scotland OR Scot OR Scots OR Ireland OR Irish OR Germany OR German* OR Holland OR Netherlands OR Dutch OR Belgium OR Belgian* OR Luxemburg* OR Benelux OR France OR French OR Spain OR Spanish OR Andorra OR Andorran* OR Portugal OR Portuguese OR Italy OR Italian* OR San Marino OR Malta OR Maltese OR Switzerland OR Swiss OR Austria OR Austrian* OR Liechtenstein OR Czech OR Slovakia OR Slovakian* OR Monaco OR Monegasque* OR Estonia OR Estonian* OR Latvia OR Latvian* OR Lithuania OR Lithuanian* OR Baltic OR Hungary OR Hungarian* OR Poland OR Polish OR Russia OR Russian* OR Ukrain* OR Belarus OR Moldova OR Moldovan* OR Armenia OR Armenian* OR Romania OR Romanian* OR Bulgaria OR Bulgarian* OR Greece OR Greek* OR Albania OR Albanian* OR Balkan* OR Slovenia OR Slovenian* OR Croatia OR Croatian* OR Macedonia OR Macedonian* OR Serbia OR Serbian* OR Montenegro OR Montenegrin* OR Kosovo* OR Mediterranean* OR Europe OR European* OR “the EU”) OR AB (Sweden OR Swedish OR Swede* OR Norway OR Norwegian* OR Denmark OR Danish OR Dane* OR Scandinavia OR Scandinavian* OR Finland OR Finnish OR Iceland OR Icelandic OR United kingdom OR British OR Brit OR Brits OR England OR English OR Wales OR Welch OR Scotland OR Scot OR Scots OR Ireland OR Irish OR Germany OR German* OR Holland OR Netherlands OR Dutch OR Belgium OR Belgian* OR Luxemburg* OR Benelux OR France OR French OR Spain OR Spanish OR Andorra OR Andorran* OR Portugal OR Portuguese OR Italy OR Italian* OR San Marino OR Malta OR Maltese OR Switzerland OR Swiss OR Austria OR Austrian* OR Liechtenstein OR Czech OR Slovakia OR Slovakian* OR Monaco OR Monegasque* OR Estonia OR Estonian* OR Latvia OR Latvian* OR Lithuania OR Lithuanian* OR Baltic OR Hungary OR Hungarian* OR Poland OR Polish OR Russia OR Russian* OR Ukrain* OR Belarus OR Moldova OR Moldovan* OR Armenia OR Armenian* OR Romania OR Romanian* OR Bulgaria OR Bulgarian* OR Greece OR Greek* OR Albania OR Albanian* OR Balkan* OR Slovenia OR Slovenian* OR Croatia OR Croatian* OR Macedonia OR Macedonian* OR Serbia OR Serbian* OR Montenegro OR Montenegrin* OR Kosovo* OR Mediterranean* OR Europe OR European* OR “the EU”	1,490,938
#5	1# AND 2# AND 3# AND 4#AND 5#	132
Limiters	Date of Publication: 20000101–20,231,231	105
Web of Science		
1#	Elder OR older OR senior OR old people OR aged OR older adult OR pensioner* (topic)	5,330,714
2#	(TI = (chinese OR china)) AND TI = (migrant* OR immigrant* OR diaspora)	4,278
3#	(AB = (chinese OR china)) AND AB = (migrant* OR immigrant* OR diaspora)	9,225
4#	#2 OR #3	10,499
#5	(TI = (Sweden OR Swedish OR Swede* OR Norway OR Norwegian* OR Denmark OR Danish OR Dane* OR Scandinavia OR Scandinavian* OR Finland OR Finnish OR Iceland OR Icelandic OR United kingdom OR British OR Brit OR Brits OR England OR English OR Wales OR Welch OR Scotland OR Scot OR Scots OR Ireland OR Irish OR Germany OR German* OR Holland OR Netherlands OR Dutch OR Belgium OR Belgian* OR Luxemburg* OR Benelux OR France OR French OR Spain OR Spanish OR Andorra OR Andorran* OR Portugal OR Portuguese OR Italy OR Italian* OR San Marino OR Malta OR Maltese OR Switzerland OR Swiss OR Austria OR Austrian* OR Liechtenstein OR Czech OR Slovakia OR Slovakian* OR Monaco OR Monegasque* OR Estonia OR Estonian* OR Latvia OR Latvian* OR Lithuania OR Lithuanian* OR Baltic OR Hungary OR Hungarian* OR Poland OR Polish OR Russia OR Russian* OR Ukrain* OR Belarus OR Moldova OR Moldovan* OR Armenia OR Armenian* OR Romania OR Romanian* OR Bulgaria OR Bulgarian* OR Greece OR Greek* OR Albania OR Albanian* OR Balkan* OR Slovenia OR Slovenian* OR Croatia OR Croatian* OR Macedonia OR Macedonian* OR Serbia OR Serbian* OR Montenegro OR Montenegrin* OR Kosovo* OR Mediterranean* OR Europe OR European* OR “the EU”)) OR AB = (Sweden OR Swedish OR Swede* OR Norway OR Norwegian* OR Denmark OR Danish OR Dane* OR Scandinavia OR Scandinavian* OR Finland OR Finnish OR Iceland OR Icelandic OR United kingdom OR British OR Brit OR Brits OR England OR English OR Wales OR Welch OR Scotland OR Scot OR Scots OR Ireland OR Irish OR Germany OR German* OR Holland OR Netherlands OR Dutch OR Belgium OR Belgian* OR Luxemburg* OR Benelux OR France OR French OR Spain OR Spanish OR Andorra OR Andorran* OR Portugal OR Portuguese OR Italy OR Italian* OR San Marino OR Malta OR Maltese OR Switzerland OR Swiss OR Austria OR Austrian* OR Liechtenstein OR Czech OR Slovakia OR Slovakian* OR Monaco OR Monegasque* OR Estonia OR Estonian* OR Latvia OR Latvian* OR Lithuania OR Lithuanian* OR Baltic OR Hungary OR Hungarian* OR Poland OR Polish OR Russia OR Russian* OR Ukrain* OR Belarus OR Moldova OR Moldovan* OR Armenia OR Armenian* OR Romania OR Romanian* OR Bulgaria OR Bulgarian* OR Greece OR Greek* OR Albania OR Albanian* OR Balkan* OR Slovenia OR Slovenian* OR Croatia OR Croatian* OR Macedonia OR Macedonian* OR Serbia OR Serbian* OR Montenegro OR Montenegrin* OR Kosovo* OR Mediterranean* OR Europe OR European* OR “the EU”)	5,181,332
#6	(TI = (high-income countr*)) OR AB = (high-income countr*)	18,029
#7	#5 OR #6	5,194,847
#8	TS = (end-of-life OR palliative OR dying OR pension OR care OR ageing OR illness OR frailty OR disease*)	10,405,513
#9	#1 AND #4 AND #7 AND #8	352
Limiters	Publication date 2000-01-01-2023-12-31	338

### Inclusion and exclusion criteria

The inclusion criteria were: (1) Studies including or about populations of older Chinese immigrants or studies focusing on their use of health/social care services, (2) Chinese immigrants in Europe, their relatives, and related health/social care professionals, (3) Qualitative peer-reviewed studies published in English, and (4) publications from 2000 to 2023 to ensure that the data captured both historical developments and contemporary insights from the time when the search was conducted. Older Chinese immigrants were defined as (1) self-defined by the person in question, (2) defined as such in the study, or (3) persons aged 60+ years. Exclusion criteria were: (1) studies conducted geographically outside of Europe, (2) non-qualitative research designs and literature reviews, (3) intervention studies, and (4) studies without ethical reasoning.

### Selecting, appraisal, and extracting relevant data

**Figure 1:**
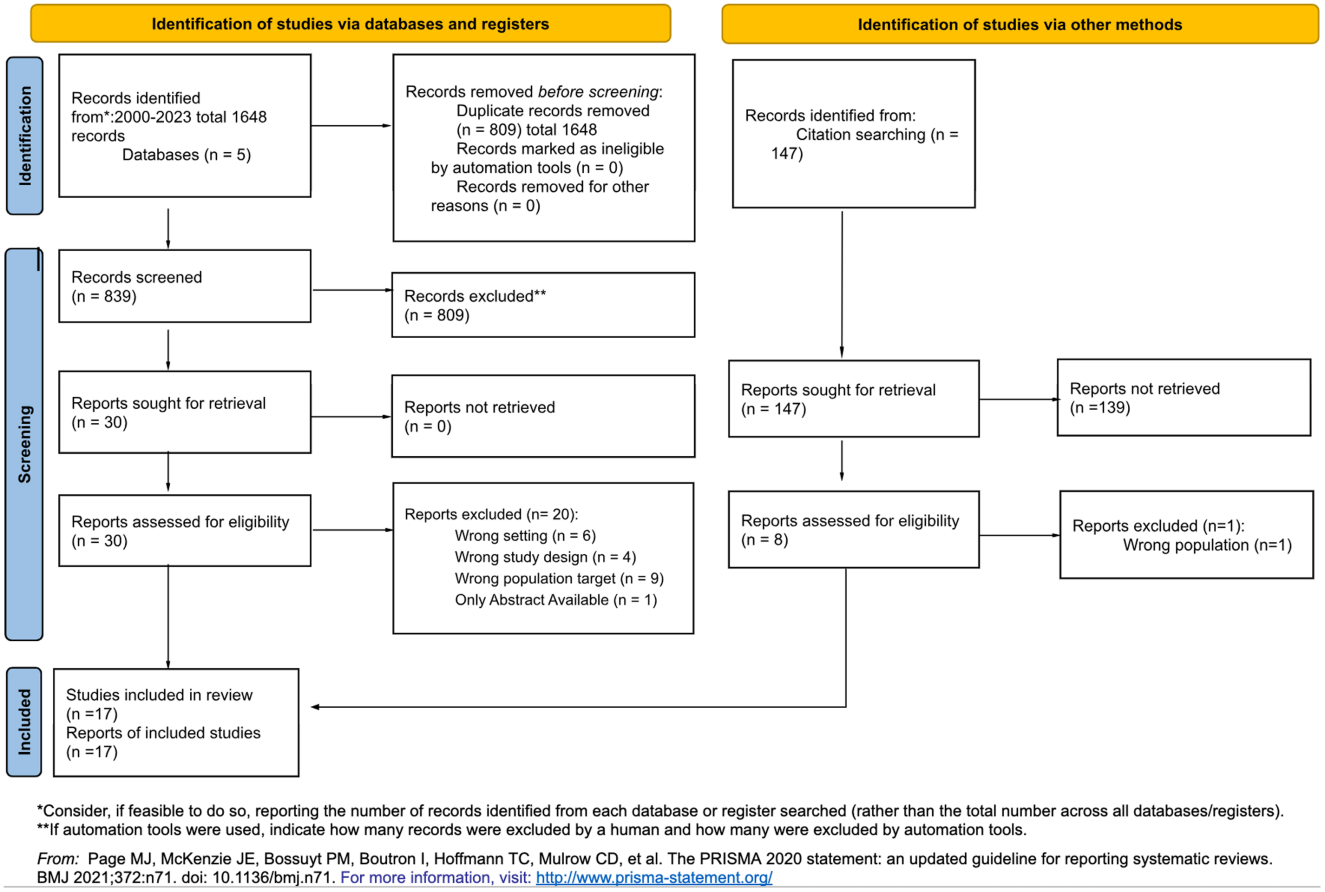
PRISMA flow diagram.

The literature search was conducted by an experienced university librarian. The initial search retrieved 842 publications, which were transferred to Covidence software for the screening process (40). The entire publication selection process was conducted collaboratively by two junior researchers (HX and CF). In case of disagreement in the screening processes, the publications that received different votes were listed in Covidence without displaying the individual votes. The two junior researchers then reassessed these publications with a fresh perspective and discussed them in collaboration with two of the senior researchers (SG and SS) until consensus was reached. According to the inclusion and exclusion criteria, ten eligible studies were found during the screening process. After the citation pearl search in Scopus, seven supplementary articles were included. A total of 17 articles were included, as shown in the screening process in the PRISMA flow diagram (Figure 1). The included publications are marked with an asterisk * in the references. The Critical Appraisal Skills Program (CASP) qualitative study checklist was used to appraise the quality of included publications by the two junior researchers (HX and CF) and no disagreements regarding study quality were found (106). No publications were excluded based on lack of methodological rigour in this process.

Inspired by the Cochrane Consumers and Communication Review Groups’ data extraction template (41), a structured data extraction spreadsheet was created including: (1) Authors, (2) Year, (3) Country, (4) Journal, (5) Study period, (6) Study design, (7) Sample size, (8) Study population, (9) Theory and concepts, and (10) Results. A selection of the extracted data is presented in Table 2. The extracted data was checked for accuracy by all authors.

**Table 2:** Study characteristics.

Author(s) year of publication study location	Aim(s) of the study	Method(s) (time period)	Study populations’ characteristics	Main findings
Chau and Yu (61) United Kingdom	To document Chinese-origin elders’ diverse migrant histories, cultural backgrounds and attitudes to both ‘traditional’ and Western health-care practices.	Study design: Case study Analytical strategy: Family culture based culturagram assessment tool Time period: N/A	Three persons immigrated to the UK (ages: 65, 65, 72)	The culturagram rapidly identified similarities and differences among three Chinese older adults in their support needs and healthcare preferences and beliefs. Those older adults with no legal civil status could not access all the National Health Service (NHS) services and had to rely on Traditional Chinese Medicine and some over-the-counter medicines to cope with their illness. They all mastered good English and believed in western medicines and were likely to be willing and able to use the mainstream health-care services. They all lived alone and found it difficult to engage informal interpreters to help them use services provided by the NHS.
Cheung et al. (47) Netherlands	To explore the perception of Chinese-Dutch young adults regarding providing care tasks for their parents and whether these are expressions of filial piety, as they have been living in Chinese households in a Western society.	Study design: Semi-structured interviews Analytical strategy: Thematic analysis Time period: 2017 May–July	Fifteen Chinese-Dutch young adults (Age range: 18–25)	From the perspectives of young adults, some older immigrants depended on their children or grandchildren to share information about local services and arrange health care services due to the language barriers. Young adults found that most older immigrants prioritised the centrality of family and that some older immigrants had voiced openness towards the use of nursing homes.
Cook (48) United Kingdom	To explore the welfare citizenship experiences of older women who migrated in later life to England, either as refugees or as post-retirement migrants.	Study design: Secondary data analysis study consisting of life-story interviews and focus groups Analytical strategy: Thematic analysis Time period: 2000–2002	Ninety-six women from five ethnic communities living in UK: black Caribbean, white British, Chinese, Irish and Somali (Age range: 50 years or older)	The older adults (aged 60–80) were more likely to be reluctant to openly criticise service provision for the Chinese community, which can be explained partly by fear of losing services and partly by their different cultural expectations. The older adults had relatively low expectations of welfare services. Chinese women’s lack of English proficiency posed problems for accessing welfare rights and services and placed significant limitations on their mobility and their relationships with their families and social networks. These Chinese women stressed the need to improve the quality of language interpretation and translation services in the welfare context. Some Chinese women depended on their families for interpretation, resulting in loss of autonomy and being a burden on their family. Some of them thus turned to local communities and cultural groups for support. Chinese community networks were needed to enable the realisation of welfare citizenship and social participation among migrant communities.
Flynn and Wong (49) United Kingdom	To explore the interplay between different employment barriers faced by one group facing significant employment barriers: older migrants.	Study design: Participatory Action Research, including semi-structured interviews Analytical strategy: Thematic analysis Time period: N/A	Eight Chinese immigrants from Wai Yin society in the UK (Age range: 55–73)	For older self-employed migrants, major social and economic disruptions may paraphrase their retirement plans. Many self-employed immigrants’ states that the work they did was physically demanding. Older migrants identified four main barriers to continued employment, including language, discrimination, their lack of employment history owing to years spent in either self- employment or precarious work, as well as the lack of planning for extended working life due to disruption of their life trajectories.
Irvine et al. (50) United Kingdom	To examine the factors affecting the social care experiences of physically disabled people from Chinese backgrounds in England.	Study design: In-depth semi-structured interviews and focus groups Analytical strategy: Thematic analysis Time period: July 2012–February 2013	Twenty-six people from a Chinese background with a physical impairment who had received social care from adult services in the previous 6 months (Age range: 19–69)	Most older participants were not aware of personal budgets that blocked the accessibility of social care. The personal budget system was generally considered complicated, which limited access to those who were aware of the system. The social services lacked personalisation, so many older participants avoided using available services to maintain their individuality. Cultural values, such as food habits, had significant implications for the needs of social care. The challenge of language differences hindered service uptake. Self-reliance created reluctance to seek outside help and deterred access to personal budgets.
Lee (46) United Kingdom	To examine how some older adult Chinese tenants in a cluster of housing schemes in the north of England differed in their perception, consciousness and management of time.	Study design: Ethnographic study with participant observation and interviews Analytical strategy: Rhythmanalysis Time period: June 1997–July 1998	Three informants: Popo (92 years old), Ah-Ngoh (over eighty years old), Onn-Sum (nearly 80 years old)	Older migrants had learned to apply and adapt skills from how they previously marked time to cope with modern-day living in a foreign urban context. Daily routines for older adults could be structured or unstructured, depending on the rhythms of their health and sleep patterns. While some older people had accepted the rhythm of retirement and were happy to do as much or as little depending on the circumstances.
Li et al. (59) United Kingdom	To explore Cantonese-speaking older Chinese migrants’ knowledge, attitudes and expectations regarding mental illness.	Study design: Semi-structured interviews Analytical strategy: Grounded Theory and in-depth content analysis Time period: N/A	Eight older Chinese people who had migrated from Hong Kong and China and who had lived in Britain for more than 10 years. (Age range: 61–92)	The older adults described mental illness as abnormal nerves, abnormal behavior, changed mood, changed personality, and idioms of “others”. They generally had a fear of mental illness, including meeting people with mental illness. Some older adults also interpreted mental illness as normal life stress, pressures associated with job loss, and lack of love in the family. Some of them did not construe dementia and Alzheimer’s disease as mental illnesses but as an old people’s matter.
Liu et al. (56) United Kingdom	To explore Chinese Elders’ experiences of and attitudes towards the provision of health services in primary care.	Study design: Open-ended in-depth interviews Analytical strategy: Grounded Theory approach Time period: July 2008–June 2009	Thirty-three first generation Chinese older adults in the UK (Age range: 60–84)	Older Chinese adults were inclined to present to General Practitioners (GPs) only when health concerns were perceived as serious. The serious health concerns were considered as being beyond their ability to self-manage. Many of the older adults tended to adopt self-management strategies rather than follow professional advice. This was mainly due to communication difficulties, poor understanding of the advice doctors gave, and the way that Chinese patients interpreted and used the advice they were given. Older adults reported that the purpose of contacting doctors was to obtain medicines. They presumed that once medication had been prescribed their symptoms would be cured, and they believed that they could self-manage their health, usually without further GP or other medical follow up.
Liu et al. (57) United Kingdom	To explore behaviors and attitudes towards exercise among older Chinese immigrants in the UK to provide insights into the health of Chinese populations in the UK and elsewhere.	Study design: In-depth semi-structured interviews Analytical strategy: Grounded Theory Time period: July 2008–June 2011	Thirty-three first generation Chinese older adults in the UK (Age range: 60–84)	Older adults’ self-managed exercise based on cultural perceptions of health and ingrained Chinese values. Professional support and information were lacking and relied on folk norms rather than person-centred recommendations for healthy living. Inappropriate exercise regimes could act as a substitute for seeking health-related advice when exercise was often used as a self-monitored barometer to assess their perceived health status.
Liu et al. (55) United Kingdom	To explore experiences of using health and care services among older Chinese immigrants in England, including barriers encountered and the coping strategies they utilised to address their circumstances.	Study design: Focus group interviews and semi-structured individual interviews Analytical strategy: Grounded Theory Time period: August 2011–May 2013	Forty-four older Chinese adults (age range: 60–80), and 15 supporters (i.e., family and friends, public sector workers, and staff from community-based Chinese organisations) (age range: 22–53)	Many older Chinese immigrants were challenged in knowing about and in accessing healthcare services. Their difficulties were attributed to language barriers, lack of information and practical support, and emotional and cultural issues regarding use of health and care services. The supporters who could facilitate older Chinese immigrants’ access to services had different backgrounds, namely family and friends, public sector workers and staff from community-based Chinese organisations. The defining attributes of these supporters were: bilinguality, bicultural, multifunctionality and accessibility.
Pan et al. (45) Netherlands and Belgium	To explore older Chinese migrants’ activity participation experiences from the perspective of Confucianism, the cornerstone of Chinese culture.	Study design: In-depth interviews Analytical strategy: Thematic content analysis Time period: N/A	Twenty-one older Chinese migrants in the Netherlands and seven in Belgium (age range: 60–88)	The four principles of Confucianism, including hierarchical relationships, family system, benevolence, and emphasis on education, had positive and negative effects on older Chinese immigrants’ activity participation. Hierarchical relationships promoted formal organisational participation, yet concurrently divided the Chinese community into smaller subgroups within the community. Older Confucianist immigrants prioritised taking care of their grandchildren, resulting in less time to participate in outdoor activities. Benevolence restrains older Chinese immigrants from political participation while encouraging them to attend community meetings. Emphasis on education helped older Chinese immigrants overcome feelings of loneliness.
Russo (60) United Kingdom	To explore how social inequalities shape the meaning of hope of Chinese people in the UK.	Study design: In-depth life history interviews Analytical strategy: Capabilities approach and intersectionality analysis Time period: 2009–2010	Twenty-two Chinese people in the UK with psychiatric diagnosis (Age range: 25–85)	For some older adults, negotiating between attempts to expand life and efforts to avoid relapse was a balancing act. Some tried to engage with the labour market. However, having reached retirement age, with the chances of being employed further decreased by ageism, some of the older adults continued to try and improve employability in other ways, such as regular attendance at the Job Centre. Having cautious pursuit of hope was the strategy to manage expectations.
Speed et al. (58) United Kingdom	To develop an under- standing of the concerns of older Chinese people with cardiovascular disease (CVD) including their management of health-seeking events with primary care services.	Study design: Focus group interview Analytical strategy: Grounded Theory Time period: N/A	Twenty-eight older Chinese people living within the local community of a “Chinatown” in England with a CVD diagnosis. (Age range: 60+ years)	Many older adults compared the services they accessed for CVD treatment in the UK to those available in China, and found the treatments they were receiving were insufficient. They reported having low expectations of primary care services and preferred to cope with symptoms themselves or to use secondary care or emergency services. All of which meant that they often dealt with symptoms until they became unmanageable or unbearable. Many of the older adults used informal sources of information and sought support from other Chinese people rather than healthcare professionals. Problems with health literacy, communication, and mismatched expectations were described.
Tang and Pilgrim (52) United Kingdom	To provide qualitative evidence from the experience of Chinese service users in the UK to expand the literature on the use of intersectionality analysis in research on the mental health of ethnic minority groups.	Study design: Repeated in-depth life-history interviews Analytical strategy: Thematic analysis Time period: N/A	Twenty-two people who themselves as Chinese and had a psychiatric diagnosis (Age range: between 30–70 years)	Many older adults found that isolation was often accumulated from their experience of a restricted social network. Placing their faith in their children as a source of contentment was a common strategy to endure the hardship. Some older adults were able to access help from Chinese community centres, yet they found it difficult to build trusting relationships with others, revealing the division within Chinese communities. While support from community centres helped them maintain daily living, an underlying loneliness remained. Some older adults found that living on their own and deteriorating physical health as major sources of stress. Not trusting people was a self-protection against the further actual or imagined malign intentions of others. Yet, a sense of self-protection strategy was counterproductive because it restricted their social network for help-seeking.
Tang (51) United Kingdom	To explore the experience of Chinese mental health service-users in the United Kingdom and discuss what a Community Development (CD) approach might entail for their recovery.	Study design: Repeated in-depth life history interviews Analytical strategy: Thematic analysis Time period: 2009–2010	Twenty-two mental health service-users who identified themselves as Chinese, and had a psychiatric diagnosis (Age range: 25–85)	For older adults, mental health discrimination intersects with racial and age discrimination puts them in a vulnerable position, especially in job hunting. Some older adults who had mental health crises at a young age had to work in low-skilled manual jobs where working conditions were poor due to lack of educational qualifications. Poor working conditions and inadequate occupational safety measures further inflict physical damage on the older adults.
Yeung et al. (54) United Kingdom	To examine the factors affecting their experiences of social care. The paper also seeks to determine their levels of satisfaction with social care so that areas for service improvement could be identified.	Study design: semi-structured, individual interviews and focus group interviews Analytical strategy: Thematic analysis Time period: 2012–2013	Twenty-six Chinese people with physical impairment and were in receipt of social care services (age range: 19–69)	Older adults with physical disabilities were struggling to manage daily challenges and perform their social roles before they received support from social care. Feeling uncertain about their entitlement to services and being unfamiliar with the procedures involved impede older participants’ help-seeking. Some older adults encountered language barriers to accessing social care. Some were satisfied with the social services they received; others expressed discontent with uncaring attitudes of social care workers. Despite the input from social care, some older adults’ needs were not adequately met. Thus, their families remained active in providing care for them. Many older adults relied on day services provided by Chinese welfare organisations to meet their social and dietary needs.
Yeung et al. (53) United Kingdom	To examine how Chinese populations make sense of the experiences of mental distress, and how this understanding influences their pathways to mental health care.	Study design: In-depth interviews with open-ended questions Analytical strategy: Thematic analysis Time period: 2010–2011	Fourteen Chinese immigrants with mental health problems (MHPs) (age range: 25–64); Sixteen family members (age range: 42–80)	For some older adults, their description and presentation of their discomfort were predominantly in a physical form. Feelings of psychological and mental distress such as sadness and anxiety were not communicated to medical professionals. Some older adults found that they had limited alternatives but to follow the medication regime. Despite the unpleasant side effects of the medication, most participants continued to take the medication to avoid compulsory treatment and admissions to hospital. Older adults with multiple relapses and admissions to hospital were more likely to search for alternative remedies to manage the distress brought forth by MHPs.

### Strategy for data analysis

The analytical synthesis of data consisted of two parts: (1) a descriptive numerical summary analysis presented as ‘Characteristics of the studies’, and (2) a synthesising, thematic analysis inspired by Braun and Clarke (42) and Bettany-Saltikov and McSherry (43). NVivo 12 computer software was used as assistance to organise and analyse the data, serving as a preliminary codebook (44). As a first step, all publications were read several times to facilitate familiarisation with the material, with particular focus on the results (42). Second, the results’ sections were initially coded in NVivo, with the codes being reorganised according to the current review’s aim by the two junior researchers (HX and CF). The codes and materials were discussed with and reviewed by the third senior researcher (RJAG) to ensure consistency and reliability. Third, the initial sub-themes were constructed based on similarities and differences of the coded material (42), and similar sub-themes were synthesised into themes. Fourth, the themes were reviewed and further developed in a consensual process of analysis amongst all authors to ensure their clarity and alignment with the study’s aim. While pursuing reflexive dialogues, the authors went back and forth between the empirical data, the constructed themes, and the current review’s aim to ascertain that the themes appropriately reflected the empirical material (42). Finally, all themes and sub-themes were defined, refined, named, and reviewed (42). The included studies were described, integrated, and synthesised through interpretations of the included articles to generate new interpretive understandings of everyday life in ageing among older Chinese immigrants in Europe, in line with Bettany-Saltikov and McSherry’s (43) suggestions. The analysis process thus included a movement towards higher abstraction levels and synthesis.

## Results

### Characteristics of the studies

In total, 17 publications based on 14 studies were included. The publications originate from the United Kingdom (*n* = 15), Netherlands (*n* = 2), and Belgium (*n* = 1), where one study was conducted in both Netherlands and Belgium (45). Sixteen publications used interviews and/or focus groups to collect data, and one publication used qualitative observation as a data collection method (46). Most articles used thematic analysis (47–54), other used grounded theory (55–58), content analysis (45, 59), rhythm analysis (46), and intersectionality analysis (60). Six publications were based on three separate studies, with three, respectively, separate populations: the publications of Irvine et al. (50) and Yeung et al. (54) were based on a shared population, as were Liu et al.’s (56) and Liu et al.’s (57) publications and Tang and Pilgrim’s (52) and Tang’s (51) publications. The 17 publications’ total population included 341 older Chinese immigrants, 16 relatives (family members and friends), and 15 health/social care professionals. The included older Chinese immigrants had self-diagnoses and/or medical diagnoses of physical impairment, cardiovascular disease, and/or a psychiatric diagnosis. Psychiatric diagnoses were included in four publications (51–53, 60), namely depression, bipolar disorder, paranoia, anxiety disorder, and schizophrenia. The publications described various limitations, such as outdated data, small sample sizes, limited scale of population recruitment, and weakness in sampling strategies (45–47, 49, 50, 53–56, 58, 59, 61). Moreover, findings from some publications might not consider some groups of older adults, for example, those who were isolated (45, 53). Based on the outcomes of the CASP checklist (106), all selected publications were deemed to have appropriate methodological rigour (Table 3).

**Table 3:** CASP qualitative study appraisal.

	Section A: Are the results valid?						Section B: What are the results?			Section C: Will the results help locally	Scores
	1. Was there a clear statement of the aims of the research?	2. Is a qualitative methodology appropriate?	3. Was the research design appropriate to address the aims of the research?	4. Was the recruitment strategy appropriate to the aims of the research?	5. Was the data collected in a way that addressed the research issue?	6. Has the relationship between researcher and participants been adequately considered?	7. Have ethical issues been taken into consideration?	8. Was the data analysis sufficiently rigorous?	9. Is there a clear statement of findings?	10. How valuable is the research?	
Chau and Yu (61)	Yes	Yes	Yes	Yes	Yes	Cannot tell	Cannot tell	Yes	Yes	Yes	8/10
Cheung et al. (47)	Yes	Yes	Yes	Yes	Yes	Cannot tell	Yes	Yes	Yes	Yes	9/10
Cook (48)	Yes	Yes	Yes	Yes	Yes	Yes	Cannot tell	Cannot tell	Yes	Yes	8/10
Flynn and Wong (49)	Yes	Yes	Yes	Yes	Yes	Yes	Yes	Yes	Yes	Yes	10/10
Irvine et al. (50)	Yes	Yes	Yes	Yes	Yes	Yes	Yes	Yes	Yes	Yes	10/10
Lee et al. (46)	Yes	Yes	Yes	Yes	Yes	Yes	Cannot tell	Yes	Yes	Yes	9/10
Li et al. (59)	Yes	Yes	Yes	Yes	Yes	Yes	Yes	Yes	Yes	Yes	10/10
Liu et al. (56)	Yes	Yes	Yes	Yes	Yes	Yes	Yes	Yes	Yes	Yes	10/10
Liu et al.(57)	Yes	Yes	Yes	Yes	Yes	Yes	Yes	Yes	Yes	Yes	10/10
Liu et al.(55)	Yes	Yes	Yes	Yes	Yes	Yes	Yes	Yes	Yes	Yes	10/10
Pan et al.(45)	Yes	Yes	Yes	Yes	Yes	Yes	Cannot tell	Yes	Yes	Yes	9/10
Russo (60)	Yes	Yes	Yes	Yes	Yes	Yes	Yes	Yes	Yes	Yes	10/10
Speed et al. (58)	Yes	Yes	Yes	Yes	Yes	Yes	Yes	Yes	Yes	Yes	10/10
Tang and Pilgrim (52)	Yes	Yes	Yes	Yes	Yes	Yes	Cannot tell	Yes	Yes	Yes	9/10
Tang (51)	Yes	Yes	Yes	Yes	Yes	Cannot tell	Cannot tell	Yes	Yes	Yes	8/10
Yeung et al. (54)	Yes	Yes	Yes	Yes	Yes	Yes	Yes	Yes	Yes	Yes	10/10
Yeung et al. (53)	Yes	Yes	Yes	Yes	Yes	Yes	Yes	Yes	Yes	Yes	10/10

### Everyday life as an older adult mirrored the life lived

Older Chinese immigrants’ daily routines in terms of exercise, activity, and self-health promotion practices were partly rooted in lifelong habits and partly inspired by cultural beliefs (46, 53, 55–58, 60, 61). Early life experiences had tempered older Chinese immigrants’ current life choices and life rhythms (46, 56, 57, 60). Some older immigrants, especially those living in sheltered housing, tended to arrange their daily lives in a steady, familiar, and accustomed way, with an attempt to integrate traditional lifestyles into the rhythms of modern life, even though some older immigrants’ daily living revolved around medication or was limited by medical conditions (46, 57). Maintaining familiarity and clarity in daily routines provided older Chinese immigrants with a sense of control in their lives, surrounded by unfamiliar languages, environments, or cultures (46, 56, 57, 60).

Some older Chinese immigrants attached great importance to health promotion and had unique insights into personal health promotion strategies (46, 53, 56–58, 60, 61). Exercise played an essential role in their daily routines and lifestyles, as physical activity was commonly considered to prevent age-related physiological deterioration (46, 56–58, 61). Those who exercised regularly in their daily routines often had clear personal opinions about exercise principles that were most beneficial for themselves, accumulated over their lifetime and in the lifestyle they had adhered to Chau and Yu (61), Lee (46), Liu et al. (66. 67), and Speed et al. (58). Some older Chinese immigrants considered frequent, low intensity exercise was suitable for their age-related physical conditions, while others used the ability to perform vigorous exercise as a self-assessment of health (57, 58). However, the reliance on exercise for detection and treatment of health and disease may lead to their reluctance to seek help due to the notion that exercise was more beneficial than medication (57, 58). Furthermore, some older Chinese immigrants’ distinctive health beliefs influenced by western medicine and traditional Chinese medicine (TCM) were derived from their specific culture and life experiences (53, 56–58, 60, 61). Although western medicine was considered more advanced and reliable in treating serious or acute illnesses, some older immigrants embraced TCM-based methods, for instance, TCM dietary therapy, traditional Chinese mind and body exercises, and herbal formulations, to relieve symptoms, prevent diseases, and promote health (53, 56–58, 61). Therefore, some older Chinese immigrants were accustomed to using a combination of western medicine and TCM for disease treatment and health promotion (53, 56, 61). However, some older Chinese immigrants’ claimed that most of their TCM knowledge came from accumulated folk norms rather than professional sources (57, 58).

### Work and working conditions as significant for ageing

Some older Chinese immigrants faced challenges in relation to work, retirement, and ageing, shedding light on how the intricate interplay of immigration and social positions influenced the ageing process (46, 49, 51, 52, 60). Older Chinese immigrants’ life trajectories and current socioeconomic status contributed to different views on the importance of work in their everyday lives during old age (46, 51). Those who were satisfied with their current jobs or had experienced stable and fulfilling employment throughout their lives were inclined to continue working in old age (46, 51). Many of the working older immigrants viewed work not only as an indispensable means of enhancing self-worth by contributing to society and providing themselves with financial resources, but also as a means of mitigating the cognitive decline associated with ageing (46, 51).

Five publications indicated that most older Chinese immigrants with work experience in the host country were often in the Chinese catering industry, owning or being involved in the family catering business or being employed by others (46, 49, 51, 52, 60). Although working in the Chinese catering industry brought many of these older Chinese immigrants a sense of financial security, it meanwhile contained multiple work-related stressors, such as demanding physical work environments, challenging interpersonal relationships, and limited employers’ support, particularly for those with disabilities or health problems (46, 49, 51, 52, 60). Some older male Chinese immigrants who worked in physically demanding jobs often developed physical impairment and disability as they aged (49, 51). Older Chinese immigrants who experienced significant hardship in their work environments were often more susceptible to social isolation, which is a common source of varying degrees of mental health problems (46, 52, 60). Moreover, lack of local language skills, lack of education and qualification, and lack of extensive social networks in the host countries—factors often seen as the cultural markers—prevented Chinese immigrants from accessing mainstream employment, i.e., industries that were typically dominated by the host country’s population (49, 51, 52, 60). Hence, to secure stable employment, they often remained confined to jobs with poor working conditions, such as those in the Chinese catering industry (49, 51, 52, 60). The sense of entrapment can contribute to social isolation, particularly for those already experiencing mental health issues (49, 51, 52, 60).

For self-employed older Chinese immigrants, saving enough money for their children’s education and for their own retirement expenses were often decisive factors in their retirement plans (49). However, the macroeconomic vulnerabilities of self-employed businesses may disrupt older immigrants’ retirement plans, leading many to extend their working life, either actively or passively (49, 60). Some older Chinese immigrants’ perceived that working into old age was often negatively interpreted in Chinese communities as a sign that older people were less prepared for retirement (49). For older Chinese immigrants still seeking work near retirement age, employment opportunities further declined due to age discrimination in the labour market (49, 60). Furthermore, for older Chinese immigrants with mental health problems, discrimination related to mental health was a common factor obstructing their employment opportunities (51, 60). Additionally, many older Chinese immigrants with mental health issues had difficulty finding a permanent job, but nevertheless felt the need to disclose their mental health history to potential employers (51, 60).

### Cultural complexes that shape social identities

Older Chinese immigrants, as described in many publications, often adhered to traditional Confucianism cultural values in how they perceived and dealt with interpersonal interactions, including within the family system, leading to disinclination for social participation and help-seeking, as well as intergenerational conflicts (45–49, 51–53, 59, 61). The principles of social relations followed by these older Chinese immigrants under the influence of Confucianism, i.e., hierarchical relationships and benevolence, often contrasted with western norms of equality (45). Some older Chinese immigrants restrained their participation in political activities as they feared to undermine social harmony advocated by traditional Chinese culture (45). Nonetheless, some older Chinese immigrants emphasised the importance of informal community networks for promoting social participation and welfare access (45, 46, 48, 51, 61). However, the contradictions between different manners for establishing social relationships inspired by different cultures hindered some older Chinese immigrants’ participation in social activities (45, 52). Different expectations on social relationships and language barriers led to frustration and confusion among older Chinese immigrants, many of whom tended to quit mainstream social activities with local people and turned to Chinese communities for a sense of social belonging (45, 46, 48, 51, 61).

The Chinese community played a substantial role in Chinese immigrants’ everyday lives, particularly for those with limited local language skills. The Chinese communities provided not only social opportunities for people with similar cultural or immigrant backgrounds but also favourable resources for accessing formal and informal welfare through information exchange (45, 46, 48, 51, 61). However, some older Chinese immigrants with mental health issues experienced exclusion or divisions within Chinese communities and thus had difficulty forming intimate and trusting relationships with people in the communities (51–53). Older Chinese immigrants dealing with mental health issues manifested a pattern of distancing themselves from the community to avoid gossip or stigma, similar to trends observed in the broader Chinese community (48, 51–53). Owing to the fact that mental health issues were highly stigmatised in Chinese societies, many older Chinese immigrants with mental health issues and their families tried to conceal personal or family history of mental health issues from the outside world through social avoidance (51–53, 60). For many older Chinese immigrants, family was often perceived as the main source of support, socialisation, and connection to the outside world (53, 60). Feelings of marginalisation among older Chinese immigrants with mental health issues further reduced their sense of self-identity and adversely affected their mental health (53, 60).

Older Chinese immigrants and their families considered the family system, influenced by Confucianism, as emphasising mutual dependence among family members (45, 47, 52, 61). Self-reliance advocated by traditional Chinese culture led to the reluctance of some older Chinese immigrants to seek help outside of the family, partly due to family values of mutual dependence and partly due to cultural beliefs of self-sufficiency (48, 50, 52, 54, 55). Often, “self” referred not only to the older Chinese immigrants themselves but to the whole family. Some older Chinese immigrants viewed family as a close-knit network where each member had a moral duty to care for one another and where mutual sacrifice was a norm (45, 47, 52, 59, 61). The conservative marriages and family lives entailed gender inequalities in terms of family structure and care responsibility, where women were often expected to bear the disproportionate burden of caring duties within the family, including caring for the younger generations, their husband, and the older generations of the family (45, 52). However, the conservative family concept induced gender-inequality consequences, causing women to lose opportunities for social participation and threatening their mental health (45, 52).

Some older Chinese immigrants additionally believed that children, as primary sources of their social interaction and connection, were the centre of a family. Even the decision to immigrate was in many cases made for the sake of their children, which was sometimes seen as a sacrifice by the older Chinese immigrants (45, 47, 52). Thus, some of these older Chinese immigrants had underlying expectations that their children would take care of them (45, 47, 52, 61), while others tended to choose future care arrangements at their own discretion (46, 48, 51). Some older Chinese immigrants recognised that their different expectations for family dynamics stemmed from the process of cultural acceptance and integration between the country of origin and the country of immigration (46, 48, 61). However, life-long family-oriented value was a major factor in social isolation in old age, contributing to loneliness, particularly in families with intergenerational cultural differences or conflicts (45–47, 52, 59, 61). Moreover, some older Chinese immigrants expressed hope for their children to improve social mobility through education and to integrate into mainstream society (49, 52, 61).

### Immigrants’ social position as significant for encounters with health and social care professionals

Older Chinese immigrants, as noted in many publications, voluntarily or involuntarily sought different pathways to meet their increased social/health needs while having complex feelings about their encounters with health/social care professionals (47, 48, 50–59, 61). Some older Chinese immigrants faced various barriers and challenges in accessing health/social care services, reflecting the social positions embedded in their immigration background (47, 48, 50–59, 61). A lack of awareness of welfare entitlements prolonged some older immigrants’ navigation of the health/social care systems, the situation compounded by a lack of language skills and instrumental barriers to services (48, 50–56, 61). Despite the availability of interpretation support from social networks and services, older immigrants’ language barriers hampered communication with health/social care professionals, which negatively affected their relationship with professionals and satisfaction with the services (48, 50, 54, 58, 61). Some older immigrants were even frustrated by the lack of direct interaction with health/social care professionals due to the involvement of interpreters (48, 50, 51, 54–56, 61). Some older Chinese immigrants and health/social care professionals stated that informational, cultural, emotional, and instrumental support provided by family members and social networks effectively helped older immigrants cope with daily life, including accessing health/social care services (47, 48, 50, 51, 54, 55, 58). Meanwhile, some older Chinese immigrants persisted in relying on informal care from family members and social networks due to previous negative experiences with health/social services or rumours, while others tended to access formal health/social care due to concerns about burdening family members and their social networks (47, 48, 50, 51, 54–56, 58). Nevertheless, some older Chinese immigrants expressed satisfaction with the health/social care they received, partly because of relatively low expectations on welfare services and partly because of concerns about losing existing services (48, 54, 56, 58).

Cultural differences significantly affected older immigrant’s expectations regarding health/social care, influencing the older adults’ views on well-being, their understanding of healthcare systems, and their beliefs about the roles of family and community in caregiving. These varying expectations sometimes led to some older immigrants questioning the fairness of healthcare equality (48, 51, 54, 56, 58, 61). Progressive acculturation influenced older Chinese immigrants’ perceptions of encounters with health/social care professionals, while older immigrants’ encounters in turn changed or reinforced their attitudes towards health/social care systems and western medicine (48, 50, 54–56). From some older Chinese immigrants’ perspectives, conventional health/social care services were not always able to accommodate different cultural practices, thus raising the need for culturally specific care services to meet their cultural and linguistic needs in health/social care (48, 50, 51, 54–56).

## Discussion

The review’s findings indicate that experiences and perceptions of becoming old as a Chinese immigrant is a dynamic process, which is in line with Johansson et al. (16). Instead of seeing ageing processes as individual endeavours, these processes are influenced by multiple factors, including institutional structures such as established systems and organisations in society, and complex interpersonal and social dynamics (16, 21). To grasp these complexities surrounding the life course of older Chinese immigrants requires reflections that go beyond stereotypes and generalisations (62, 63). The discussion will focus on three main findings, namely (1) ageing as an immigrant was not only a dynamic process influenced by social, interpersonal, and institutional factors accumulated in their life trajectories, but also a process of constructing social and cultural identity in their new homeland, (2) older Chinese immigrants’ health beliefs were influenced by both TCM and western medicine, in which physical activities played an essential role in structuring everyday lives, and (3) potential or actual barriers and stigma in relation to health/social care may lead older Chinese immigrants to be reluctant or marginalised in face of their care needs.

The results showed that older immigrants’ ageing process in their new homeland mirrored their life course while facing new challenges of acculturation and integration. Bourdieu (64) argues that people’s habitus—or practical sense—is ruled by fundamental structures in life and contexts and operates beneath consciousness and will. In this way, habitus influences and shapes individuals’ perceptions, preferences, and actions, constructing different socialisation forms and societal positioning (64). This main finding indicated that variations in older Chinese immigrants’ everyday lives were largely shaped by their perceived social status and life trajectories including immigration. However, the length of the Chinese immigrants’ stay and their social backgrounds were not considered in many of the included studies. This could have contributed to a deeper understanding of this group’s lives as older adults and calls for future studies that take such factors into account. Previous research suggests that a person’s early-life circumstances, which are heavily influenced by socioeconomic factors, can have cumulative effects on the ageing process, and lead to a decline in functions over time (65). Culture also plays a significant role in shaping perceptions of ageing, with individualistic cultures predicting more positive attitudes towards older adults (66). Given that, the main finding indicates that older Chinese immigrants’ self-perceptions of ageing may also be influenced by socialisation processes in their host country. A previous study shows that immigration often brings with it a loss of social status (67). Immigrants who are accustomed to being part of the dominant culture in their country of origin are often viewed as a minority when moving to a new country (i.e., their new homeland) (68). Consequently, people tend to categorise themselves and others based on various characteristics such as race, ethnicity, age, and gender, leading to distinctions between “insiders” and “outsiders” in society (69). The above-mentioned main finding indicated that older Chinese immigrants as ‘outsiders’ might refer to ‘mainstream’ activities in society as a denotation of the ‘insiders’. Several studies point out that the ‘insiders’ and ‘outsiders’ distinctions often arise from negative stereotypes and racial biases against immigrants, whereby people who are similar to the native majority in terms of ethnic background, religion, dress, or accent are considered similar and ‘insiders’, otherwise they will be seen as different and ‘outsiders’ (69, 70). Additionally, studies show that older immigrants commonly perceive downward mobility in social hierarchy compared to the host population due to language barriers, differences in educational attainment and qualification recognition, and limited social networks and resources (71). It is, however, worth noting that social categorisation, such as ‘insiders’/‘outsiders’, is prevalent not only among minority ethnic groups but also among other groups based on social position in society, age, and gender. Thus, for older Chinese immigrants, the intersectionality of these social categorisations, such as social position, age, gender and immigrant background, can lead to combined and interdependent consequences of discrimination (72).

Moreover, the results showed that tensions could be seen between approaches stemming from more collectivist societies in the East and individualistic societies in the West, with older Chinese immigrants being prone to adapt to and adopt values differing from their original ones. Older immigrants navigating between two different cultures and their respective social norms may pose challenges to socialising in the host society (73). Similar bicultural identity negotiations have been observed among South Asian and Middle Eastern immigrants in Europe (74). Previous studies have shown that older Chinese immigrants, particularly first-generation immigrants in Europe, tend to maintain close ties with their ethnic cultural customs and rarely integrate into the host culture (75, 76). Consequently, older immigrants may maintain cultural values and beliefs that differ from those of younger generations, who are influenced by individualistic norms prevalent in western societies (77). This generational divide can create tensions in family dynamics, especially concerning caregiving duties and expectations, which can intensify social isolation among older immigrants (77, 78). Studies suggest that acculturation pathways play a crucial role in the positive adjustment of immigrants in distinct ways, in which ethnic and host cultures can coexist while remaining unique to the individual (79, 80). Nguyen and Benet-Martínez (81) point out that maintaining ethnic and host culture ties can lead to better psychological and sociocultural adjustment among older immigrants. As noted by Ward and Szabó (73), adapting to the host society may require significant changes in beliefs, behaviors, and identities based on cultural differences. Studies argue that although people from different backgrounds may have different habits, cultures, or beliefs, sharing common norms and social rules may contribute to a sense of belonging and identity (82). The immigrant’s subjective sense of belonging coupled with the actual rights and responsibilities as an actual citizen can create a connection between the immigrant and the host population, further deepening the immigrant’s sense of belonging and identity in the host society (83). Additionally, immigrants’ work experience is a key indicator of social integration, as employment fosters social participation, language acquisition, and network development within the host society. It also contributes to retirement security through access to pensions and financial stability in later life (84, 85). Therefore, this main finding pointed to the importance of social belonging, whereby individuals identify themselves with characteristics and values, as an essential component of social integration.

Furthermore, another main finding was that older Chinese immigrants’ health beliefs entailed both TCM and western medicine elements, but their health promotion strategy as an incorporated part of many Chinese older immigrants might imply western political ideology. Previous research highlights that physical activity is often associated with ideas about promoting health and prolonging lifespan, such that the capability to perform physical exercise is often being seen as emblematic of the will to live (86, 87). Several studies suggest that TCM, along with other eastern medicinal practices, emphasises the importance of somatic integrity and emotional self-mastery in achieving good health, which can be accomplished by the flow of stretching and swaying movements rather than strenuous muscle motions (88, 89). However, some studies take a different approach to ageing and physical activity. For example, Jette and Vertinsky (90) examine how western biomedicine overlooks the impact of culture and social environment on exercise choices in multicultural societies. This suggests that there is limited knowledge about how individuals from diverse cultures perceive and engage in body practices like exercise, and how these practices are rooted in different knowledge systems (90). Malcolm et al. (91) show how neoliberalism fosters an ideology of healthism, promoting forms of exercise which diverge from health maximising behaviors. Neoliberalism extends differences in physical activity across social positions with embedded health inequalities. This aligns with a newly published article by Aamann and Dybbroe (107), who find that discourses of active ageing create distinctions between older adults where different practices are recognised as either ‘good’ or ‘bad’ by healthcare professionals. In sum, this illustrates how discourses of ‘active ageing’ might contribute to the emergence of class-based differentiation among older adults, regardless of their ethnic origin (107).

Moreover, the results showed that potential or actual barriers and inequalities in health/social care settings might hinder active health-seeking behaviors among older Chinese immigrants. This pattern has also been observed among South Asian, Middle Eastern, and African immigrants in European countries, where language barriers, mistrust of healthcare institutions, and culturally insensitive services lead to lower healthcare utilisation (92). In western societies, healthcare systems are grounded in the foundational principle of equality, often referred to as egalitarian discourses. These discourses uphold the belief that individuals are accountable for their social position and behavior, irrespective of their circumstances (93). This may obscure the unequal power dynamics between patients and healthcare professionals, unconsciously overlooking the structural and social inequities that impact people’s health (93–95). In public healthcare discussions, patients are often described as active subjects rather than passive objects. Consequently, they are expected to adopt a proactive role in preserving and restoring their health (86). This complexity of opposing elements serves as the underlying structure shaping widely accepted ideas and social dynamics (64). Added to this, mental health challenges may increase the burden through experiences of (self) stigma and more acute experiences of isolation and alienation (96, 97), although attribution theories pertaining to mental illness may affect or moderate people’s attitudes towards those affected (98). Ingvarsdotter (99) argues that in cross-cultural contexts, overemphasis on the role of culture in mental illness coping mechanisms and resilience may be viewed as justifications for reducing the allocation of medical resources. As highlighted by previous research (33, 34, 94), the current results underscore that, despite the presence of formal health/social care services, not everyone had equitable access to these services. Older Chinese immigrants and their families expressed the necessity for health and social services that could adeptly cater to and respect their cultural and linguistic needs. Moreover, older Chinese immigrants’ health-seeking behaviors exhibit different patterns based on individual interpretations of multiple cultural beliefs and community influence. In this case, the language aspect introduces an additional layer of complexity, as limited language proficiency often creates communication barriers for older Chinese immigrants when interacting with health/social care professionals (100, 101). Studies highlight that communication barriers, along with differences in values, perceptions, expectations and ways of expressing and behaving between immigrants and health/social care professionals, may result in distrust and ‘othering’, which illustrates the complexity of building trust in intercultural healthcare settings (100). Altogether, the results showed the importance of what Antonovsky (102) named a sense of coherence, based on individuals’ experience of comprehensibility, manageability, and meaningfulness, which can affect levels of resiliency in life, also in relation to (im)migration (103). The cultivation of cultural humility among health/social care workers but also amongst researchers, whereby one examines one’s own beliefs and cultural identities and learn about other cultures through self-reflection and self-critique (104), may help minimise health disparities through enhanced sensitivity and awareness (105).

The current study has strengths and limitations. The quality of the included studies was high, ensuring the findings’ quality and trustworthiness and their relevance for the review’s aim. The literature search was performed after consultation with and support from an experienced university librarian, which has strengthened the accuracy of identification of relevant studies. The review did not specify a specific age or number of years as a definition for old age, which is why the current review covers different perceptions, references, and self-definitions of old age and ageing processes. This may be regarded as a strength in line with the understanding that ageing is not about fixed numbers of years, but an aspect that must also be understood in relation to personal experiences and social circumstances (21). The analysis process requires the authors’ constant reflection on their subjectivity and interpretations pertaining to the included publications’ contents (108). This was achieved through regular discussions and mutual agreement amongst all authors throughout the analysis process, strengthening the study’s rigour and credibility. As limitations, this review includes only peer-reviewed articles and studies in the English language, excluding potentially relevant grey literature, pre-print studies, and studies in other languages. Studies published in other languages and studies with other designs, such as longitudinal studies which were not found in the database searches, may also have offered additional nuanced perspectives, particularly in understanding how ageing trajectories and migration experiences evolve over time. The analysis did not adopt a life course approach as data on individuals’ past life histories and backgrounds was unavailable. However, integrating this approach could have offered valuable insights into how previous life events and migration trajectories shape their ageing experiences in various ways. The findings were presented without specifying the perspectives of older Chinese immigrants, relatives, and health/social care professionals because the data did not fully differentiate between the viewpoints of each group. The included study’s origins were limited to only three European countries although the review intended to target all European countries, the vast majority of which were from the United Kingdom. It is unknown if the included studies’ results mirror life conditions for the Chinese immigrant population in the named countries, and it is questioned if this can be generalised/transferred to other contexts.

## Conclusion

The current review provides a picture of the daily lives of older Chinese immigrants in Europe, involving their perceptions of the processes of ageing in their new homeland. The results highlight the complex interplay among social, interpersonal, and institutional structures that underlie the dynamic ageing process among older Chinese immigrants in their new homelands. The various patterns of interaction with health/social care professionals reflect the uniqueness of acculturation processes that are influenced by individual life trajectories and social circumstances. The results point to the need for future research to explore the intergenerational interactions in terms of acculturation and its influence on older Chinese immigrants’ ageing processes. Future research is also needed to focus on the impact of social inequalities on older Chinese immigrants’ ageing experiences, including health and health conditions. International research collaborations and mixed-methods studies may also contribute to a broader and deeper understanding of older Chinese immigrants’ experiences of daily life and ageing across a diversity of geographical locations. Finally, the study has policy implications that emphasise the need for culturally responsive healthcare, social services, and employment support to better meet the unique ageing experiences of older Chinese immigrants. Promoting community integration and inclusive policy-making is essential to reducing social isolation and ensuring equitable access to resources.

## Data Availability

The original contributions presented in the study are included in the article/supplementary material, further inquiries can be directed to the corresponding author.
